# Are we good enough? A measurement for Information Technology Service Quality (ITSQ) in higher education institutions in Saudi Arabia

**DOI:** 10.1371/journal.pone.0277265

**Published:** 2022-11-17

**Authors:** Romana Aziz, Ala Saleh Alluhaidan

**Affiliations:** Department of Information Systems, College of Computer and Information Sciences, Princess Nourah Bint Abdulrahman University, Riyadh, Saudi Arabia; King Saud University, SAUDI ARABIA

## Abstract

In the present research, we aim to confirm factors for measuring the perceived quality of Information Technology (IT) services within a higher education context. The perceived quality of IT services is complex and is often measured by multi-dimensional constructs, which requires designing a sufficiently valid scale. Drawing upon literature and expert input, this study has identified 5 dimensions of IT service quality. Using an empirical study, we prove that perception of IT Service Quality (ITSQ) has a dimensional structure and can be measured using a 44-item scale which has been satisfactorily validated. A Confirmatory Factor Analysis (CFA) is applied to confirm the relationship between the items and dimensions. The findings of this study present a scale for measuring ITSQ within higher education.

## Introduction

In rapidly changing technology era, the information technology service quality (ITSQ) has become a major determinant of organizational success. Improving the service quality of information technology has become a primary factor in the successful execution of the strategic plan of any organization including higher education institutions. A well-established high quality ITSQ is a key to enhance the quality of higher education institutions. Most Higher Education Institutions (HEI) are striving to make the latest advanced technology available for their staff, faculty, and students. Establishing a standard process for measuring ITSQ is still considered a challenging endeavor [1, 2].

**Fig 1:**
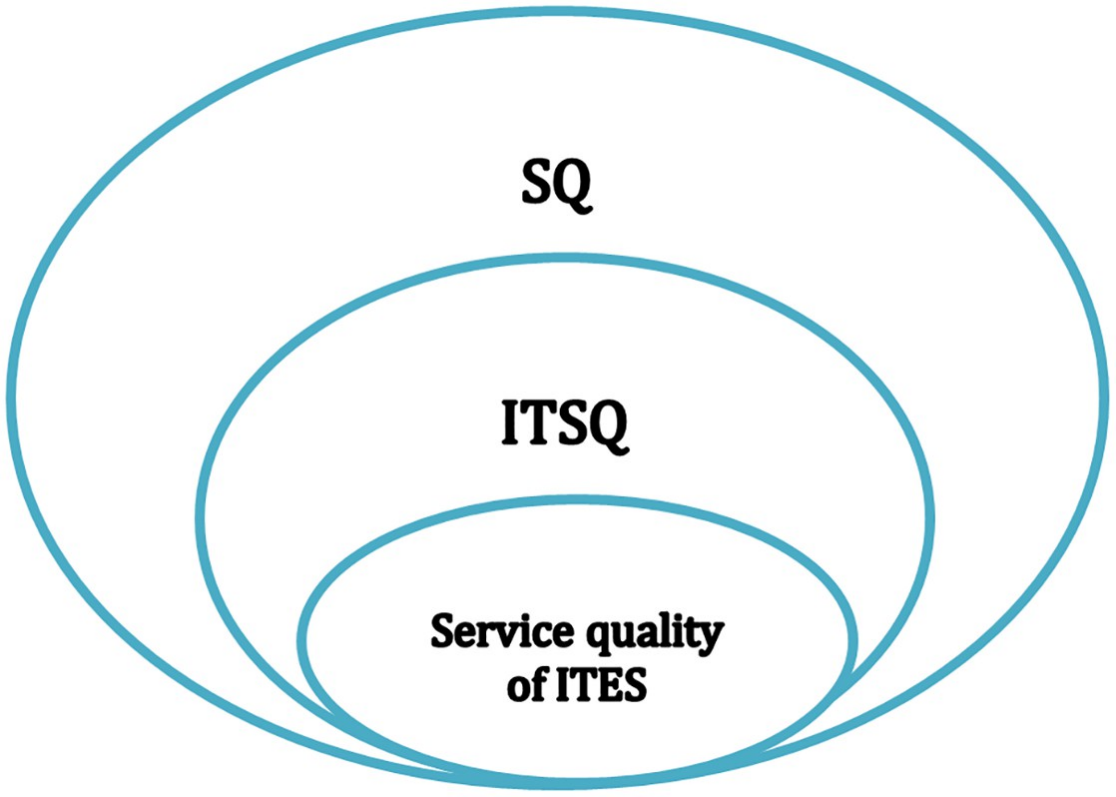
Hierarchy of service quality concepts.

ITSQ may be measured by the stakeholder’s needs or what is perceived as a good practice for IT service. The goal of evaluating ITSQ is to have an indication of service performance, identify service problems to enact improvements, and guarantee optimum service for all. In the extant literature, there are three related streams of research: 1) service quality, 2) IT service quality, and 3) quality of IT-enabled services, see Fig 1. The existing literature covers many domains such as banks, airlines, healthcare, …, etc.

In the area of higher education, a lot of work has been done regarding the quality of higher education with respect to teaching and learning. Yet, there is a need to develop and validate a scale to measure IT service quality in higher education [1]. This is because IT is playing a dominant role in delivering services in higher education which makes it an integral component for assessing stakeholder satisfaction with provided services. Researchers have proposed some instruments for measurement of service quality in areas like healthcare, higher education, banking, …,etc. In this work, we have adapted and extended the existing knowledge for developing and validating an instrument for measurement of ITSQ in higher education institutions.

Looking closer into the “quality” definition in the education context, some have proposed it as high quality in administration, equipment, facility, and instructions. In [3], the authors refer to quality as helping students acquire graduate skills and what employers are looking for. The paper [3] is focused on undergraduate students, academic staff, and employers’ perspectives. Questionnaires data was collected from over 300 students, around 30 staff, and 17 employers. Also, qualitative data were gathered from students in the form of focus groups. Results revealed that employers highly appreciated graduate personal qualities, while students highly valued quality of education and learning, feedback, and staff.

The quality of education has improved with provision of higher quality IT services. Dissemination of information, differentiation in teaching techniques, and diversity in delivery methods have increased and improved as IT services prosper. The research paper [4] investigates the quality of IT-Enabled Services (ITES) in the higher education institutes in Saudi Arabia. Specifically, a mixed research method was used to gain information from two models in higher education institutions: -public and private universities. Results show the quality of IT-enabled services within a public institution is better than in private institution.

With improved quality in IT services in higher education, services become cheaper, faster, and more precise. This consequently helps in reducing university workload while automating most processes. With enforced usage of IT within an organization, students will be obligated to improve their communication abilities, computer skills, team working attitudes, and collaborative learning tendencies [2]. This paper helps in measuring ITSQ within the higher education sector. It synthesizes and presents factors of the quality of ITSQ. Addressing the following question: What are the current factors in measuring information technology service quality in higher education.

With the advancement in technology and services provided, there is a need to measure user satisfaction and standardize the process of such a measurement. We chose a higher educational system as a target domain, as the available literature of this type of measurement needs to be updated. Therefore, the present study addresses this gap by developing a reliable and valid scale for measuring ITSQ.

The paper is organized as follows, first the literature review is covered, then methodology and results are explained. The last two sections are discussion and conclusion.

## Literature review

Standardizing services to maintain satisfaction is a goal in many fields. For example, in healthcare domain, an article investigated critical satisfaction factors in the healthcare domain regarding IT and information quality, system quality, service quality, professional maturity, and personal innovativeness were suggested as the umbrellas [5]. This study provides valuable insights for decision-makers in the healthcare domain. In fact, assessing service quality of healthcare management from the patient’s perspective is essential in the evaluation and implementation of corrective procedures. The paper [6] provides a systematic review of Multiple Criteria Decision-Making (MCDM) measures that are used in healthcare service for quality evaluation. MCDM measures are used widely in healthcare to reach decisions. Over 42 publications were covered from 33 journals spans over 12 years from 2004 to 2016. Yet, the scale is designed with an eye of a healthcare perspective and our targeted domain is higher education.

### Quality in HE

The higher education institutions are facing an ever increasing pressure to offer high-quality services to students, staff, and faculty, therefore, there is a need to measure how well the services are rated by consumers. The previous research has been focused only on ITES in higher education which we chose to have as a starting point for our work to develop and test an instrument for IT service quality in higher education.

Considering how quality is assessed, European countries agreed that quality is measured internally and not by comparison as the standards varied [7] for organizations as well as their goals. Yet, autonomy is one of the aspects that is emphasized for quality in Europe. One of the most commonly known methods for measuring service quality is SERVQUAL, which is a comprehensive measurement instrument. SERVQUAL started with ten dimensions then decreased to five dimensions: reliability, responsiveness, assurance, empathy, and tangibles [8]. The scale in this case is neither tailored for IT nor has been customized for higher education.

A literature review of the service quality (SQ) from articles published from 1984 to 2017 was covered here to understand issues involved in service quality [1]. In general, the quality was discussed from conceptualization and operationalization perspectives. Results show different categories of services indicate different quality measures. Healthcare, manufacturing, banking, information technology, and higher education are the top sectors investigated in the literature. “More than 60 models of the SQ have been identified. Service-driven capabilities may be structured along adaptation with strategic drivers and imperatives, learning and alignment, and problem structuring [1].”

In [2], authors present the impact of information systems in enhancing service quality of higher education institutions. In this study, they modeled information system from the perspective of hardware, software, human, information, and knowledge that are integrated into teaching, learning, administration, research, and community engagement.

For evaluating quality of service in higher education at Marmara and Niğde Omer Halisdemir Universities, a screening model was used. The model consists of 28 items and 6 factors. The study was conducted during the 2016–2017 academic year and data of 886 students was collected. Gender, grade, university, and academic success were used as personal variables. The results show that females were higher than males with respect to the academic reputation and institutional image. Additionally, “the perceptions of students in 3rd grade were higher than students in 4th grade according to academic reputation, institutional image, offered diploma programs, and physical opportunities.” [9] This study is focused on students’ appreciation of the quality needed as they graduate.

Higher education service quality management and improvement were discussed in [10]. The paper instruments Schneider and Bowen’s model, which contains three layers of service organizations and service quality management and enhancement methods, on higher education institutions. Results show quality of service given to the students by each employee who has interaction with the students is greatly dependent on top HE management and different departments in the institution. Paper presents practical implications and recommendations on how to administer and improve service quality in higher education.

Students’ satisfaction and loyalty with their university have been investigated in [11] under conditions of highly competitive environment between higher education institutions. Data were collected through an online questionnaire from 14,870 students at the University of Pisa. The results show valuable insight on how teaching and lectures as well as course organization are the key factors of students’ satisfaction and students’ loyalty.

Paper [12] studies how to measure satisfaction with an emphasis on sustainability considering variables such as groups (family, teachers, and pupils). After confirming factors of the instrument, reliability was calculated through confirmatory factor analysis. Results show significant differences between groups. Therefore, the role performed by a quality education with sustainability equally includes teachers, students, and surrounding communities. This role incorporates effective methods into teaching, methods for encouraging members of the educational community and providing knowledge, skills, attitudes, and necessary values. The characteristics and experiences of individuals with their education are all critical here as indicated by the study.

A study by [13], aims to measure academic quality improvement in higher education through examining the quality of medical courses in 4 medical universities and assessing them according to the 9 dimensions of the Academic Quality Improvement Program (AQIP) model. AQIP scales contain these dimensions: a) support learning, b) clear achieving goals, c) understanding needs, d) appreciating people, e) leading and communicating, f) maintain operations, g) measuring effectiveness, h) constant improvement, i) building cooperative relationships. The study was conducted in Iran choosing Isfahan, ShahidBeheshti, Tehran, and Iran universities. The sample consists of academic members, graduate students, as well as scientific board partners. Although four universities were relatively ranked high on 9 dimensions of the AQIP model, there was a difference between students and scientific board members on how they judge the academic quality improvement.

In reviewing several quality constructs, their application, success, and flaws, in higher education services [14], is scoped to discuss total quality management (TQM), Kaizen, Six Sigma, Lean and Lean Six Sigma (LSS), comparing their value and their inadequacy in absorbing quality within higher education. Specifically, the focus of the research is identifying the success and shortcomings of several quality constructs in higher education services. Findings show a demand for a more comprehensive instrument with complete constructs to measure the quality of service within the HE context.

### ITES

Technology-enabled services (TESs) are perceived differently by customers and [15] investigates passengers’ perceived importance of various airline TESs. Established TES, network access, and new TES are three categories of TESs while the two dimensions of Technology Readiness (TR) are optimism and innovativeness. TR was found significantly coupled with the perceived importance of TESs. With higher levels of optimism, established TESs was rated as very important while network access and new TESs were rated highly on innovativeness and regarded as more important. The relationship between TR dimensions and the perceived importance of TESs was more apparent in passengers of low-cost airlines than in customers of full-service airline [15].

IT-Enabled Services (ITES) are embedded in many fields and higher education is now picking up on the importance of having higher quality in ITES. The paper by [16] identifies 17 factors for measuring the quality of ITES in higher education universities in Saudi Arabia. Factors are: “accessibility, delivery of teaching, efficiency, information quality, inter-operability, privacy, security, response time, service reliability, service usability, site design, system integrity, user support, customization, functionality, trust, and usefulness.” [16] Authors used an analytic hierarchy process to extract factors from publications. We used this article in fact as a starting point for building the instrument for ITSQ.

In order to form the scale for IT service quality factors, we review the articles that covered the recent advances and measures [1, 4, 16]. Findings show a list of suggested factors [4, 16] while another research evaluated services against the latest technology [1]. Overlapping factors and updating services requirements were the primary sources for factors and items presented in this work.

## Methodology

Our study is centered on a specific problem and is real-world practice-oriented. The main focus is to develop and validate an instrument that can be used by higher management to measure the quality of IT services in higher education. Therefore, in accordance with these characteristics of our work, we used a mixed-methods approach based on pragmatism in our research [17]. There were two phases in our research, a qualitative phase was followed by a quantitative phase. The participants were required to sign a written informed “Consent Form” which explained the purpose of the research. The study included only adults. The two phases are depicted in Table 1 and explained in the following paragraphs.

**Table 1:** Methodology.

Phase	Type	Steps
1	Qualitative	Identification of dimensions from literature review
		Identifications for items in each dimension from literature review
		Content validity via expert reviews
		Development of questionnaire
		Pretest of questionnaire by experts
		Questionnaire refinement
2	Quantitative	Data collection
		Data analysis: factor analysis, Average variance extracted analysis, composite reliability and fit indices calculations.
		Refinement of dimensions and items

In the first phase, an IT service quality measurement model for higher education was developed on the basis of a comprehensive review of the literature and expert interviews with academics and IT professionals. The author’s 2015 paper [16] had short-listed seventeen factors for IT-enabled services’ quality from 102 general service quality factors. On the basis of the literature review, the seventeen factors for the Quality of ITES dimension were reviewed and updated in [7]. In our current work in addition to IT-enabled services’ quality four more dimensions were identified for the IT service quality measurement model for higher education on the basis of literature review. These dimensions which were identified from the literature were found to be consistent with the findings of our interviews with academics and experts.

Moreover, items for all dimensions were identified from the literature review. The content validity of resultant dimensions and their items was ensured via subjective expert reviews and pre-survey tests. The experts’ panel consisted of two senior faculty members and one IT manager. The recruitment of experts’ panel was through email. They were asked for an interview after reviewing the initial questionnaire and having given their feedback. The interview was conducted over Microsoft Teams for 45 minutes. The interview questions included: what is their opinion in general about the questionnaire?, what are the most tangible and intangible items pertaining to ITSQ in their opinion?, and what are the items that would be good to be included in the questionnaire? Interview notes were recorded by the interviewer (one of the authors) and then discussed with the coauthor.

The items were updated in the light of expert opinion and pretest. A questionnaire was developed using five-point likert scale based on the five dimensions and identified items. The survey was tested and refined before data collection. The test was done with a panel of three IT experts. The test was done to ensure the clarity of questions and the matching of questions with factors and dimensions.

At the end of the qualitative phase, we had identified the following dimensions and items:

Quality of User support by the IT staff (9 items)
Quality of Physical Environment in IT labs/classroom (7 items)
Quality of Technical Environment in IT labs/classroom (12 items)
Quality of ITES (28 items)
IT facilities for distance learning (9 items)

The dimensions and items are given in Table 2.

**Table 2:** Identified factors and items.

Dimension	Item	Status after factor analysis
User support by the IT staff	The IT staff have the required knowledge to resolve user problems.	
(9 items)	The behavior of IT staff is polite and courteous.	Dropped
STAFF	It is easy for users to communicate problems to IT staff.	Dropped
	The IT staff provides satisfactory problem resolution.	
	The IT staff immediately addresses urgent needs	
	IT staff shows a real interest in solving problems	
	Ease to find an e-mail or phone number to contact the IT staff if there is a problem.	Dropped
	The IT staff delivers its services within reasonable time with no delays.	
	The IT staff always follows up on users problems without a need for reminders.	
Physical environment in IT labs /class rooms	Condition of lab interior furnishing	
(7 items)	Temperature level	Dropped
PHYSICAL	Cleanliness	
	Arrangement of seats in the lab	
	Fire protection safety	
	Chairs and tables	
	Placement of wires and connectors	
Technical Environment in IT labs / classrooms	Required software tools and platforms for courses are available in labs.	Dropped
(12 items)	Wide range of equipment and computers are available in labs to support courses.	Dropped
TECHNICAL	Printing facilities are adequately available.	Dropped
	Photocopy facilities are adequately available.	Dropped
	Delivery of teaching in labs is satisfactory.	Dropped
	Projectors are adequately used in teaching.	Dropped
	Smart boards are effectively used in teaching.	
	Projectors are always in a functional condition.	
	Smart boards are always in a functional condition.	
	Lab computers are always in a functional condition.	
	Fast internet connectivity is always available.	Dropped
	Lab equipment is readily usable at the start of lab session	Dropped
Quality of ITES	The ITES websites are loaded quickly into browsers.	Dropped
(28 items)	ITES the pages load at high speed during navigation	Dropped
ITES	ITES can be accessed without problem using preferred platform (browser/devices) to perform user tasks.	Dropped
	IT department always informs users when the ITES are unavailable due to maintenance tasks.	Dropped
	The ITES web sites make it easy to find what is need.	
	The ITES web sites make it easy to get anywhere on the site.	
	The ITES web sites enable users to complete a task quickly.	
	Information in the ITES web sites is well organized.	
	The ITES web sites are simple to use.	
	The ITES web sites are well organized.	
	There are no broken hyper links in the websites	Dropped
	All functions needed to perform/complete user tasks are available from the website.	
	ITES websites lets the users customize their content to serve their needs better.	
	Information contained in the ITES websites is current.	
	Information contained in the ITES websites is relevant.	
	Information contained in the ITES websites is accurate.	
	Information contained in the ITES websites is at the right level of detail.	
	Information contained in the ITES websites is in appropriate format.	
	Information contained in the ITES websites is easy to understand.	
	The ITES websites have adequate security features for user authentication.	
	The ITES websites do not share users personal information with other sites and users.	
	User data is protected from unauthorized modifications.	
	ITES websites are available all the time.	
	All components of ITES websites work properly.	
	User data in ITES websites is never lost.	
	Navigation icons in the ITES websites are consistent.	Dropped
	ITES websites have easy to understand help features.	
	Satisfaction level with the ITES	
IT facilities during distance learning	Quality of guidance provided by IT staff to solve problems during distance learning	Dropped
(9 items)	Audio quality during synchronous lectures	Dropped
DL	Video quality during synchronous lectures	
	Screen sharing during synchronous lectures	
	Ease of taking online exams	Dropped
	Communication with instructors during distance learning	
	Quality of recorded lectures	
	Ease of interacting with discussion board	
	Ease of taking online assignments	

The second phase of our research was based on quantitative methods. Once the questionnaire was developed, it was used to collect data. The purpose of the survey was to empirically test the proposed scale and to test its reliability and validity.

### Data collection

An Institutional Review Board (IRB) exception was granted by Princess Nourah bint Abdulrahman University IRB with project number [2–0304] because there is no identifiable information in our questionnaire. Upon receiving IRB exempt, the survey was conducted among a target random sample of students who are at the College of Computer and Information Sciences at Princess Nourah Bint Abdulrahman University, Riyadh, Saudi Arabia. The sample size was determined according to the requirements of factor analysis. Field [18], recommended sample size of 300 or more for the provision of stable factor analysis. The data were collected in person by visiting the classrooms and administering the survey. The students were asked to click on a web link to fill the survey form. Approximately 500 students filled the survey and after cleaning 371 usable and complete questionnaires were obtained which is well above the limit prescribed by Field [18].

### Data analysis

Field [18], recommended sample size of 300 or more for the provision of stable factor analysis. The sample size, in this case, was 371, which is acceptable for the use of factor analysis. Factor analysis is supplemented by average variance extracted analysis, composite reliability, and fit indices calculations.

### Factor analysis

Factor analysis was done for the five sets of items belonging to the five dimensions of IT service quality to confirm the relationship. Factor loading of all items was calculated to assess the extent to which the item is related to its dimension. Factor loading was calculated using the correlation coefficient for the five dimensions and their 65 items as shown in Table 1. The value 0.7 or higher factor loading represents that the factor is suitable for the dimension. Therefore, cut-off point for factor loading was chosen as 0.7 [19], although three values very close to 0.7 were retained (0.685, 0.699, and 0.695). On the basis of this criteria, 21 items were deleted from all dimensions.

The deleted items are also indicated in Table 2. The factor loading for the remaining 44 items is given in Table 3.

**Table 3:** Factor loading.

	Dimension	Item	Factor Loading
1	User support by the IT staff	The IT staff have the required knowledge to resolve user problems.	0.784
2	STAFF	The IT staff provides satisfactory problem resolution.	0.751
3		The IT staff immediately addresses urgent needs	0.767
4		IT staff shows a real interest in solving problems	0.76
5		The IT staff delivers its services within reasonable time with no delays.	0.772
6		The IT staff always follows up on users problems without a need for reminders.	0.777
7	Physical environment in IT labs /class rooms	Condition of lab interior furnishing	0.782
8	PHYSICAL	Cleanliness	0.808
9		Arrangement of seats in the lab	0.775
10		Fire protection safety	0.731
11		Chairs and tables	0.83
12		Placement of wires and connectors	0.74
13	Technical Environment in IT labs / classrooms	Smart boards are effectively used in teaching.	0.704
14	TECHNICAL	Projectors are always in a functional condition.	0.857
15		Smart boards are always in a functional condition.	0.907
16		Lab computers are always in a functional condition.	0.685
17	Quality of ITES	The ITES web sites make it easy to find what is need.	0.699
18	ITES	The ITES web sites make it easy to get anywhere on the site.	0.705
19		The ITES web sites enable users to complete a task quickly.	0.734
20		Information in the ITES web sites is well organized.	0.741
21		The ITES web sites are simple to use.	0.767
22		The ITES web sites are well organized.	0.815
23		All functions needed to perform/complete user tasks are available from the website.	0.802
24		ITES websites lets the users customize their content to serve their needs better.	0.803
25		Information contained in the ITES websites is current.	0.798
26		Information contained in the ITES websites is relevant.	0.835
27		Information contained in the ITES websites is accurate.	0.828
28		Information contained in the ITES websites is at the right level of detail.	0.782
29		Information contained in the ITES websites is in appropriate format.	0.818
30		Information contained in the ITES websites is easy to understand.	0.797
31		The ITES websites have adequate security features for user authentication.	0.741
32		The ITES websites do not share users personal information with other sites and users.	0.753
33		User data is protected from unauthorized modifications.	0.74
34		ITES websites are available all the time.	0.756
35		All components of ITES websites work properly.	0.732
36		User data in ITES websites is never lost.	0.747
37		ITES websites have easy to understand help features.	0.784
38		Satisfaction level with the ITES	0.792
39	IT facilities during distance learning	Video quality during synchronous lectures	0.695
40	DL	Screen sharing during synchronous lectures	0.738
41		Communication with instructors during distance learning	0.759
42		Quality of recorded lectures	0.78
43		Ease of interacting with discussion board	0.867
44		Ease of taking online assignments	0.816

### Average variance extracted analysis

Average Variance Extracted analysis (AVE) was used to establish construct validity. AVE analysis is shown in Table 4. The square root of every AVE value belonging to each dimension is larger than any correlation among any pair of dimensions.

**Table 4:** AVE analysis.

	STAFF	PHYSICAL	TECHNICAL	DL	ITES
STAFF	0.7685				
PHYSICAL	0.592	0.7784			
TECHNICAL	0.52	0.611	0.794		
DL	0.525	0.45	0.416	0.7777	
ITES	0.691	0.613	0.513	0.587	0.7722

### Composite reliability

Composite reliability also called construct reliability is a measure of internal consistency in scale items and is similar to Cronbach’s alpha [20]. Cut-off points for composite reliability are suggested in literature from 0.60 and above. Values for composite reliability are given in Table 5.

**Table 5:** Composite reliability.

Dimension	CR (good if >.7)
STAFF	0.896457
PHYSICAL	0.902049
TECHNICAL	0.870567
DL	0.901399
ITES	0.970085

### Fit indices

The fit indices are used to measure the degree of overall fit of a model to data. Those metrics with the numbers indicate if the model is good enough for practical purposes. Fit indices are developed based on test statistics [21]. Four fit indices were also calculated to assess factor analysis. They are given in Table 6.

**Table 6:** Fit indices.

Fit Index	Good Fit Value	Calculated Value
CMIN/DF	Good fit between 2–5	2.106
CFI	Good fit ≥.90	0.923
RMSEA	Good fit < 0.08	0.055
SRMR	Good fit <0.08	.0450

## Discussion

At the end of the qualitative phase, we had identified sixty-five items spread across five dimensions. The confirmatory factor analysis was done to confirm the relationship of items and dimensions using a factor loading cut-off point of 0.7 [19]. As a result of factor analysis, 20 items resulted in factor loading less than 0.7 and they were dropped. The number of dropped items in every dimension is:

- Quality of User support by the IT staff (3 out of 9 items were dropped)
- Quality of Physical Environment in IT labs/classroom (1 out of 7 items was dropped)
- Quality of Technical Environment in IT labs/classroom (8 out of 12 items were dropped)
- Quality of ITES (6 out of 28 items were dropped)
- IT facilities for distance learning (2 out of 9 items were dropped)

The dropped items are indicated in Table 1. The number of remaining items in the five dimensions was:

- Quality of User support by the IT staff (6 items)
- Quality of Physical Environment in IT labs/classroom (6 items)
- Quality of Technical Environment in IT labs/classroom (4 items)
- Quality of ITES (22 items)
- IT facilities for distance learning (7 items)

The factors and their items are listed in Table 2.

For the remaining items construct validity, construct reliability and four fit indices were calculated as already explained above. The purpose of these calculations was to establish the validity of the proposed scale.

Our study is one of the first empirical researches to propose a model to measure IT service quality in higher education. This research fills a gap between theory and practice of IT service quality measurement in higher education. The results show that the universities can use this model for IT service quality measurement.

The model was finalized based on data collected from female Saudi students only. It is recommended to include the perspective of the other stakeholders in a future study. The model can be further refined by repeating the study in different cultures.

## Conclusions

This paper describes a study to reveal a feasible and practical instrument to measure the level of IT service quality at a higher education institution. The experiment aims to determine elements and factors to consider when designing and implementing an IT service quality scale. Once a standard is established, measuring quality is the first step towards understanding institution services and people’s satisfaction.

The main features to measure the quality of ITQS are: user support by the IT staff, physical environment in IT labs/classroom, technical environment in IT labs/classroom, ITES, and IT facilities for distance learning. We have conducted a mixed method approach for creating and validation the scale. Our data points to benefits and promising direction of improving the quality of IT services. Our results suggest that quality is more than just giving a service, tracking, and logging. It has the potential for advancing and impacting people’s usability of technology. Our study contributes a measurement scale for IT Service Quality that could be utilized in higher education institutions especially with a major shift to online education and digital accessibility after COVID 19.

The instrument proposed and validated in this paper can help the HEIs in many ways. Institutions can use it to measure and improve their IT service quality. The instrument can also be used for guidance in case an organization wants to develop Key Performance Indicators for their IT service quality. In case an institution decides to outsource their IT services then the instrument can help in deciding the clauses of service level agreements.

### Future work and limitations

Future studies can incorporate number of claims and accessibility of web services into the scale. Also, there is a plan to implement the scale in two different organizations for comparing different sizes or different sectors such as public and private organizations. A case study also could utilize the scale to measure the improvements of services before and after.

Another suggested direction for future work is the measurement of IT service quality from the perspective of persons with disability. Accessibility principles for persons with disability have been suggested in the literature [22] and there is a need to assess IT service quality with respect to these principles.

## Supporting information

S1 File (PDF)

S2 File (DOCX)

S3 File (XLSX)

S4 File (DOCX)
