# Supplementary material for: Are we good enough? A measurement for Information Technology Service Quality (ITSQ) in higher education institutions in Saudi Arabia
Source: PLoS One. 2022 Nov 17;17(11):e0277265. doi: 10.1371/journal.pone.0277265 (PMC9671338; doi:10.1371/journal.pone.0277265)
Supplement: S2 File — (DOCX) [file pone.0277265.s002.docx]

**Study Leadership**

We are the researchers Romana Aziz and Ala Alluhaidan, faculty at college of computing and information systems at Princess Nourah bint Abdulrahman University. I am asking you to take part in our research project.

**Purpose**
The purpose of this study is to evaluate ITES services before and after COVID 19.

**Eligibility**
To take part in this study, you must be over the age of 18.

**Participation**
In this study, you will be asked to participate in a survey. During the survey, you will be asked about your general demographic information, experience with technology, IT staff, labs and classroom IT capability, and ITES features. Your participation in this study is voluntary and you are free to withdraw from the survey or the study at any time. Each survey should take about 15 minutes of your time.

**Risks of Participation**

There are no risks taking the survey. You may be offended by some of the questions in the interview or some of the information you are exposed to while answering the questions. You are free to stop the survey or your participation in the study at any time.

**Confidentiality**
The data generated from the survey will be confidential and no identifying information about you will be disclosed. All responses will be presented in summary form in any papers, books, talks, posts, or stories resulting from this study. We may share the data set with other researchers, but your identity will not be released.

**Further Information**

If you have questions regarding this study, you may contact asalluhaidan@pnu.edu.sa or raabdulaziz@pnu.edu.sa. You may print and keep a copy of this consent form.

**Consent**
Signing the form below means that you understand the information on this form, that any questions you may have about this study have been answered, and that you are eligible and voluntarily agree to participate. Refusal to sign the form will terminate the survey process.

**أصحاب الدراسة**

نحن الباحثتان رومانا عزيز والاء اللحيدان أعضاء هيئة التدريس في كلية الحاسبات ونظم المعلومات بجامعة الأميرة نورة بنت عبد الرحمن. نطلب منكم المشاركة في مشروعنا البحثي. الغرض من هذه الدراسة هو تقييم خدمات تقنية المعلومات ITES قبل وبعد COVID 19.

**يجب أن يكون عمرك فوق 18 عامًا للمشاركة في هذه الدراسة.**

**للمشاركة في هذه الدراسة**

سيُطلب منك المشاركة في استطلاع. سيتم سؤالك عن معلومات عامة خاصة بك ، والخبرة مع التكنولوجيا ، وموظفي تكنولوجيا المعلومات ، والمختبرات ، وقدرات تكنولوجيا المعلومات في الفصول الدراسية ،بالإضافة الي ميزات خدمات تقنية المعلومات (ITES ). مشاركتك في هذه الدراسة تطوعية ولك مطلق الحرية في الانسحاب من الاستبيان أو الدراسة في أي وقت. قد يستغرق كل الاستطلاع حوالي 15 دقيقة من وقتك.

**مخاطر المشاركة**

لا توجد مخاطر في إجراء الاستطلاع. لك الحرية في إيقاف الاستبيان أو مشاركتك في الدراسة في أي وقت.

**سرية المشاركة**

ستكون البيانات الناتجة عن الاستطلاع سرية ولن يتم الكشف عن أي معلومات تعريفية عنك. سيتم تقديم جميع الردود بشكل موجز في أي أوراق أو كتب أو محادثات أو منشورات أو قصص ناتجة عن هذه الدراسة. قد نشارك مجموعة البيانات مع باحثين آخرين ، ولكن لن يتم الكشف عن هويتك.

للمزيد من المعلومات او إذا كانت لديك أسئلة بخصوص هذه الدراسة ، يمكنك مراسلة asalluhaidan@pnu.edu.sa أو raabdulaziz@pnu.edu.sa. كما يمكنك طباعة نموذج الموافقة هذا والاحتفاظ بنسخة منه.

**الموافقة علي المشاركة**

يعني التوقيع على النموذج أدناه أنك تفهم المعلومات الواردة في هذا النموذج ، وأن أي أسئلة قد تكون لديك حول هذه الدراسة قد تمت الإجابة عليها ، وأنك مؤهل وتوافق طواعية على المشاركة. سيؤدي رفض التوقيع على النموذج إلى رفض المشاركة.
