# Supplementary material for: Are we good enough? A measurement for Information Technology Service Quality (ITSQ) in higher education institutions in Saudi Arabia
Source: PLoS One. 2022 Nov 17;17(11):e0277265. doi: 10.1371/journal.pone.0277265 (PMC9671338; doi:10.1371/journal.pone.0277265)
Supplement: S4 File — (DOCX) [file pone.0277265.s004.docx]

| **Consent Form**  **Study Owners**  We are in the College of Computers and Information Sy vstems at Princess Noura Bint Abdulrahman University. We ask you to participate in our research project. The purpose of this study is to evaluate IT services (ITES) before and after COVID 19.  **You must be over 18 years old to participate in this study.**  **Participation**  You will be asked to participate in a survey. You will be asked about your general information, experience with technology, IT personnel, laboratories, IT capabilities in the classroom, as well as the benefits of IT Services. Your participation in this survey is voluntary and you are free to opt out or study from the survey. At any time Each survey may take about 15 minutes of your time.  **Sharing risks**  There are no risks involved in conducting a survey. You are free to stop the survey or your participation in the study at any time.  **Share confidentiality**  The data generated by the survey will be confidential and no personally identifiable information about you will be disclosed. All responses will be presented concisely in any papers, books, talks, publications or stories resulting from this study. We may share the data set with other researchers, but your identity will not be revealed.  For more information or if you have questions regarding this study, you can write to asalluhaidan@pnu.edu.sa or raabdulaziz@pnu.edu.sa. You can also print this consent form and keep a copy of it.  **Agree to share**  Signing the form below means that you understand the information on this form, that any questions you may have about this study have been answered, and that you qualify and voluntarily agree to participate. Refusal to sign the form will result in a refusal to participate.  Yes  No |
| --- |
| **Measuring IT Servi uh,ce Quality in Higher Education**  **Questionnaire:**  **5 dimensions, 66 measures**  ***Dimension 1: Quality of User support by the IT staff***  *9 measures*  All responses will be recorded on 5 point Likert scale:  Strongly disagree  Disagree  Neutral  Agree  Strongly agree  1-M11: The IT staff have the required knowledge to resolve my problems.  2-M12: The behavior of IT staff is polite and courteous.  3-M13: It is easy to communicate my problems to IT staff.  4-M14: The IT staff provides satisfactory problem resolution.  5-M15: If I have an urgent need, the IT staff immediately addresses it.  6-M16: When I have a problem the IT staff shows a real interest in solving it.  7-M17: I Can easily find an e-mail or phone number to contact the IT staff if there is a problem.  8-M18: The IT staff delivers its services within reasonable time with no delays.  9-M19: The IT staff follows up on my problems until they are solved.  ***Dimension 2: Quality of physical environment in IT labs /class rooms***  *7 measures*  All responses will be recorded on 5 point Likert scale:  Poor  Somewhat Acceptable  Acceptable  Very Good  Excellent  10-M21: Condition of lab interior furnishing  11-M22: Temperature level  12-M23: Cleanliness  13-M24: Arrangement of seats in the lab  14-M25: Fire protection safety  15-M26: Chairs and tables  16-M27 Placement of wires and connectors  ***Dimension 3: Quality of Technical Environment in IT labs / classrooms***  *11 measures*  All responses will be recorded on 5 point Likert scale:  Strongly disagree  Disagree  Neutral  Agree  Strongly agree  17-M31: Required software tools and platforms for my courses are available in labs.  18-M32: Wide range of equipment and computers are available in labs to support my courses.  19-M33: Printing facilities are adequately available.  20-M34: Photocopy facilities are adequately available.  21-M35: Delivery of teaching in labs is satisfactory.  22-M36: Projectors are adequately used in teaching.  23-M37: Smart boards are effectively used in teaching.  24-M38: Projectors are always in a functional condition.  25-M39: Smart boards are always in a functional condition.  26-M310: Lab computers are always in a functional condition.  27-M311: Fast internet connectivity is always available.  28-M312: Lab equipment is readily usable at the start of lab session  ***Dimension 4: Quality of ITES (on campus vs distance learning mode)***  *Note: IT Enabled services (ITES) cover the entire range of organizational operations which exploit Information Technology for improving efficiency. ITES are usually delivered via internet. ITES in universities involve a wide range of services like:*   - *Learning Management System (Blackboard, Moodle etc.)* - *Library management system including access to e resources* - *Systems for admissions / registration / attendance / results (e-register / Banner)* - *Shared repositories for course portfolios and other documents*   *7 sub dimensions*  All responses will be recorded on 5 point Likert scale:  Strongly disagree  Disagree  Neutral  Agree  Strongly agree  * measures will be recorded twice for:  1. on campus teaching  2. distance learning  Seven sub dimensions (or factors)  Dimension 4 Sub Dimension A: 5 measures  Accessibility  * 29-M4A1: The ITES websites are loaded quickly into browsers.  * 30-M4A2: When I navigate through pages of ITES the pages load at high speed.  * 31-M4A4: I do not encounter any problem in accessing the ITES using my preferred platform (browser/devices) to perform my tasks.  * 32-M4A5: IT department always informs me when the ITES are unavailable due to maintenance tasks.  Dimension 4 Sub Dimension B: 6 measures  Efficiency  * 34-M4B1: The ITES web sites make it easy to find what I need.  * 35-M4B2: The ITES web sites make it easy to get anywhere on the site.  * 36-M4B3: The ITES web sites enable me to complete a task quickly.  * 37-M4B4: Information at the ITES web sites is well organized.  * 38-M4B5: The ITES web sites are simple to use.  * 39-M4B6: The ITES web sites are well organized.  Dimension 4 Sub Dimension C: 4 measures  Functionality  * 40-M4C1: There are no broken hyper links in the websites  * 41-M4C3: All functions needed to perform/complete my tasks are available from the website.  * 42-M4C4: Using the ITES lets me customize its content to serve my needs better.  Dimension 4 Sub Dimension D: 7 measures  Information Quality  * 43-M4D1: Information contained in the ITES websites is current.  * 44-M4D2: Information contained in the ITES websites is relevant.  * 45-M4D3: Information contained in the ITES websites is accurate.  * 46-M4D4: Information contained in the ITES websites is at the right level of detail.  * 47-M4D6: Information contained in the ITES websites is in appropriate format.  * 48-M4D7: Information contained in the ITES websites is easy to understand.  Dimension 4 Sub Dimension E: 3 measures  Privacy and security  * 49-M4E1: The ITES websites have adequate security features for user authentication.  * 50-M4E2: The ITES websites do not share my personal information with other sites and users.  * 51-M4E3: My data is protected from unauthorized modifications.  Dimension 4 Sub Dimension F: 3 measures  Service Reliability  * 52-M4F1: ITES websites are available all the time.  * 53-M4F2: All components of ITES websites work properly.  * 54-M4F3: My data in ITES websites is never lost.  Dimension 4 Sub Dimension G: 4 measures  Service usability (UX)  * 55-M4G2: Navigation icons in the ITES websites are consistent.  * 56-M4G3: ITES websites have easy to understand help features.  * 57-M4G4: I am satisfied with the ITES provided to me.  ***Dimension 5: Quality of IT facilities during distance learning***  *7 measures*  All responses will be recorded on 5 point Likert scale:  Poor  Somewhat Acceptable  Acceptable  Very Good  Excellent  58-M51: Quality of guidance provided by IT staff to solve problems during distance learning  59-M52: Audio quality during synchronous lectures  60-M53: Video quality during synchronous lectures  61-M54: Screen sharing during synchronous lectures  62-M55: Ease of taking online exams  63-M56: Communication with instructors during distance learning  64-M57: Quality of recorded lectures  65-M58: Ease of online communication via discussion board  66-M59: Ease of submitting online assignments |
