# Supplementary material for: Are we good enough? A measurement for Information Technology Service Quality (ITSQ) in higher education institutions in Saudi Arabia
Source: PLoS One. 2022 Nov 17;17(11):e0277265. doi: 10.1371/journal.pone.0277265 (PMC9671338; doi:10.1371/journal.pone.0277265)
Supplement: S1 File — (PDF) [file pone.0277265.s001.pdf]

Kingdom of Saudi Arabia
Ministry of Education
Princess Nourah bint
Abdulrahman University
(048)
Graduate Studies and Scientific
Research Vice- Rectorate

المملكة العربية السعودية
وزارة التعليم
جامعة الأميرة
نورة بنت عبدالرحمن
(٠٤٨)
وكالة الجامعة للدراسات العليا والبحث العلمي

IRB Registration Number with KACST, KSA: H-01-R-059

August 24, 2020
IRB Log Number: 20-0304
Project Title: How COVID 19 has impact ITES in higher education
Category of Approval: EXEMPT

Dear Dr. Ala Alluhaidan and Dr. Romana Aziz,

Thank you for submitting your proposal to the PNU Institutional Review Board. Your proposal was evaluated considering the national regulations that govern the protection of human subjects. The IRB has determined that your proposed project poses no more than minimal risk to the participants. Therefore, your proposal has been deemed ***EXEMPT*** from IRB review. Please note that this approval is from the research ethics perspective only. You will still need to get permission from the head of the department in PNU or an external institution to commence data collection.

Please note that the research must be conducted according to the proposal submitted to the PNU IRB. If changes to the approved protocol occur, a revised protocol must be reviewed and approved by the IRB before implementation. For ***any*** proposed changes in your research protocol, please submit a Request for Modification form to the PNU IRB. Please be aware that changes to the research protocol may prevent the research from qualifying for exempt review and require submission of a new IRB application or other materials to the PNU IRB. In addition, if an unexpected situation or adverse event happens during your investigation, please notify the PNU IRB as soon as possible. If notified, we will ask for a complete explanation of the event and your response.

Please be advised that regulations require that you submit a progress report on your research every 6 months. Please refer to the protocol number denoted above in all communication or correspondence related to your application and this approval. You are also required to submit any manuscript resulting from this research for approval by IRB before submission to journals for publication.

For statistical services you are advised to contact the Data Clinic at the Health Sciences Research Center (hsrc-DC@pnu.edu.sa) or the Scientific Research Center at the Deanship of Scientific Research (dsr-rsc@pnu.edu.sa) extension 30711.

We wish you well as you proceed with the study. Should you have additional questions or require clarification of the contents of this letter, please contact me.

Sincerely Yours,

جامعة الأميرة نورة بنت عبدالرحمن
مجلس المراجعة المؤسسي
Institutional Review Board (IRB)
24 AUG 2020

Prof. Omar H. Kasule Sr.
Chairman, Institutional Review Board (IRB)
Princess Nourah bin Abdulrahman University, Riyadh, KSA
Tel: +966 548867916
E-mail: irb@pnu.edu.sa
